# Workload, Usability, and Engagement with a Mobile App Supporting Video Observation of Methadone Take-Home Dosing: Usability Study

**DOI:** 10.2196/42654

**Published:** 2023-07-13

**Authors:** Bulat Idrisov, Kevin A Hallgren, Alyssa Michaels, Sean Soth, James Darnton, Paul Grekin, Steve Woolworth, Andrew J Saxon, Judith I Tsui

**Affiliations:** 1 Department of Health Systems and Population Health University of Washington Seattle, WA United States; 2 Department of Psychiatry and Behavioral Sciences University of Washington School of Medicine Seattle, WA United States; 3 Division of HIV, Infectious Diseases, and Global Medicine, Department of Medicine University of California San Francisco San Francisco, CA United States; 4 Evergreen Treatment Services Seattle, WA United States; 5 Division of General Internal Medicine University of Washington Seattle, WA United States; 6 Center of Excellence in Substance Addiction Treatment and Education Veterans Affairs Puget Sound Health Care System Seattle, WA United States

**Keywords:** addiction, direct observed therapy, health app, methadone, mHealth, mobile app, mobile health, opioid, smartphone app, substance use, usability, user engagement, user testing, workload

## Abstract

**Background:**

Methadone, a cornerstone of opioid use disorder treatments for many decades, is an essential tool for combatting the opioid epidemic. However, requirements for observing methadone dosing in person through direct observed therapy (DOT) impose significant barriers for many patients. Digital technology can facilitate remote DOT, which could reduce barriers to methadone treatment. Currently, there are limited data on the usability of such technology among patients and counselors in methadone treatment settings.

**Objective:**

The primary objective of this study was to assess the workload, usability, and engagement of a video-based DOT mobile app for patients with opioid use disorder receiving methadone treatment. The secondary objective was to assess the workload, usability, and engagement of the provider-facing app portal used by counselors.

**Methods:**

Patients (n=12) and counselors (n=3) who previously tried video DOT for methadone through a smartphone app in an opioid treatment program participated in usability testing sessions. Participants completed essential tasks for video DOT, then provided ratings of workload (NASA Task Load Index), usability (modified System Usability Scale), and engagement (modified Engagement Scale) with the core features of the video DOT program

**Results:**

Patients and counselors reported low mental, physical, and temporal demands, successful performance, low effort, and low frustration associated with activities. Patients reported high usability (mean 85, SD 9.5) and engagement (mean 3.8, SD 1.1); counselors reported moderate usability (mean 43.3, SD 17.7) and engagement (mean 2.81, SD 0.63).

**Conclusions:**

A mobile health app that facilitates video-based DOT for methadone required a low workload for patients and counselors and was highly usable for patients in an opioid treatment program; however, there are opportunities to improve usability and engagement for the counselor-facing portal.

## Introduction

Opioid use disorder (OUD) remains a major cause of mortality in the United States [1]. Methadone is 1 of 3 OUD pharmacotherapies approved by the US Food and Drug Administration but requires frequent in-person observed dosing (ie, direct observed therapy [DOT]) at a federally certified opioid treatment program (OTP) to mitigate the risks of medication diversion and overdose. The requirement of DOT can impose barriers for patients and limit access to treatment [2].

Mobile health (mHealth) technology has the potential to help reduce barriers to methadone treatment [3]. For example, smartphones allow patients to video-record themselves taking methadone at home or send messages to clinical providers, which can reduce the need for frequent visits to an OTP for DOT. However, in-person DOT remains the standard by regulation in OTPs [4].

In response to concerns about respiratory illness transmission during the COVID-19 pandemic, a large OTP agency with 3 separate sites in Washington state conducted a pilot program between April and August 2020 aimed at reducing the need for in-person DOT and doing remote screenings of COVID-19 symptoms. During the pilot, a subset of patients was invited to use the Emocha mHealth app to facilitate video-based DOT for all methadone take-home doses along with COVID-19 symptom screening completed with each video DOT submission.

In a study (Hallgren et al [5]) describing the clinical pilot, we showed that patient adherence to video DOT varied, but on average, video DOT significantly increased the number of days of observed methadone dosing and most patients received increased methadone take-home dosing privileges due to their ability to demonstrate treatment stability. However, the direct usability of the video DOT app has yet to be tested with patients or counselors in methadone treatment settings.

For this study, patients and counselors who participated in the pilot program were invited to participate in an evaluation of the app’s overall usability. We hypothesized that the mobile app would have favorable workload, usability, and engagement for patients with OUD receiving methadone treatment, including for patients who had higher versus lower adherence to the app during the original pilot program. We also hypothesized that counselors would report favorable workload, usability, and engagement for the provider-facing app portal. 

## Methods

### Study Design and Sample

Patients and counselors who participated in the original pilot program and were still receiving care or employed by the methadone treatment program were invited to participate in the usability study between May and August 2021. Patients and counselors were invited using phone calls, letters, and flyers distributed at the OTP. Recruited counselors were also encouraged to refer participants from the pilot to take part in this usability study. Efforts were made to recruit patients who in the original clinical pilot had low adherence (less than 18 video uploads), medium adherence (18-45 video uploads), and high adherence (more than 45 video uploads), defined by terciles of video uploads. Additional information describing the original pilot program and outcomes was reported by Hallgren et al [5].

### Testing Procedures

Participants completed a single usability session conducted 1-on-1 with a research coordinator in a private setting following a standardized protocol. After providing informed consent, participants completed a demographic questionnaire. The research assistant (RA) administered usability testing tasks and questionnaires and recorded data into REDCap. To complete usability testing tasks, patients either used the mobile app on a study phone or downloaded the app to their phone.

In the first session of the usability study, our RA provided verbal instructions and prompts as participants engaged in each of the tasks. This approach was chosen because, unlike a self-help app where users typically interact with the app without guidance, the Emocha app is designed for users who receive instructions from health care staff on how to complete specific tasks. Therefore, providing instructions during the usability testing accurately reflects the intended user experience and was seen as the most appropriate methodology for this study. One illustrative example of the scripts used by our RAs during the study is as follows: “Please open the mobile app and log on using the provided username and password.”

Patient participants were asked to complete 5 tasks that were determined by the study team to be the most important for successful video DOT: logging into the account, completing a COVID-19 symptom screener, uploading a video of themselves simulating methadone ingestion, sending and checking messages to a counselor, and accessing and reviewing a calendar showing methadone adherence. Counselors completed usability testing tasks on the provider-facing web portal using a study computer if the visit was in person or through their own work or personal computer if the visit was conducted remotely through Zoom. Counselor participants were asked to complete 5 tasks, that is, add a new patient, review 1 patient video, change a patient’s video regimen time and number of uploads, send and check messages, and check the patient “adherence calendar.”

The research coordinator timed each task and observed whether it was completed successfully. Participants provided NASA Task Load Index (NASA-TLX) ratings after each activity. After all activities were completed, participants completed the System Usability Scale (SUS) and User Engagement Scale-Short Form (UES-SF), described below.

### Ethics Approval

All procedures were approved by the University of Washington Institutional Review Board (review number STUDY00011142).

### Measures

#### NASA-TLX Measure

The NASA-TLX is a validated measure [6] of the cognitive workload required to complete a task. Participants self-report the mental demand, physical demand, temporal demand, performance, effort, and frustration associated with each of the 5 activities completed during the usability testing session on a visual analog scale of 0-100. An overall task load index was computed as the unweighted mean rating across all 5 activities [7]. We derived the following cutoffs to interpret mean workload: <33 for low workload, 33-66 for moderate workload, and >66 for high workload. These cutoffs were informed by Patel et al [8], who reviewed workload ratings for 21 electronic medication adherence apps and found an average workload of 50 (SD 26) with some of the least workload-heavy products having mean ratings of around 29.

#### SUS Measure

The SUS is a validated self-report usability measure [9]. It has 10 statements (5 positively framed and 5 negatively framed) that are rated on a 5-point scale completed at the end of the testing session. Total scores were calculated following standard instructions [10] to produce a total score ranging from 0 to 100, with higher scores indicating greater usability. Similar to our NASA-TLX scores, we interpreted mean scores <33 as low usability, 33-77 as moderate usability, and >77 as high usability. These cutoffs were informed by previous studies showing that the most usable medication adherence products had mean SUS scores of about 78 (SD 15) and the least usable medication adherence products had mean scores of around 28 (SD 21) [8].

#### UES-SF Measure

User engagement reflects the depth of cognitive, temporal, affective, and behavioral investment when interacting with a digital system [11] and was measured using the UES-SF [12]. The UES-SF is a 12-item self-report measure with 4 dimensions reflecting focused attention, perceived usability, aesthetic appeal, and reward factor; the latter subscale combines a felt sense of novelty, involvement, and endurability experienced while interacting with the digital system. Each question is answered on a 5-point rating scale. Following recommendations by O’Brien [11] and O’Brien et al [12], we calculated mean scores for each subscale and an overall engagement score reflecting the mean rating of all 12 items (negative engagement items were reverse coded). For this analysis, an average score higher than 3.5 would indicate high engagement.

#### Analytic Approach

Descriptive statistics characterized the patient sample. Workload, usability, and engagement measures were analyzed descriptively by computing means and 95% CIs of composite indices within the patient and counselor cohorts. Additional descriptive analyses were performed within patient subgroups who, during the original clinical pilot, had video DOT adherence that was considered low to moderate (n=8 patients) and high (n=4 patients) during the first 60 days from enrollment.

## Results

### Description of Sample

The study recruited and enrolled 12 of the 60 patients who participated in the clinical pilot (2=low adherence, 6=medium adherence, and 4=high adherence) and 3 of the 5 counselors. Table 1 describes the patient participants in the usability study. Demographics for counselors are not reported due to the small number of those participants. On average, patients were in their late 40s and most were male and White.

**Table 1:** Description of study patients who participated in the study. Demographics for counselors are not reported to preserve confidentiality, given the small sample size (n=3).

Characteristics		Patients (n=12), n (%)
**Age (years)**		
	<30	0 (0)
	30-49	5 (42)
	50-64	7 (58)
	≥65	0 (0)
**Sex**		
	Male	9 (75)
	Female	3 (25)
**Race^a^**		
	American Indian or Alaskan Native	2 (17)
	Asian or Asian American	0 (0)
	Black or African American	0 (0)
	Native Hawaiian or Pacific Islander	1 (8)
	White	9 (75)
	Unknown or another race	0 (0)
**Ethnicity**		
	Hispanic or Latino	0 (0)
	Not Hispanic or Latino	12 (100)
	Unknown	0 (0)
Homeless^b^		0 (0)

^a^Nonexclusive category.

^b^Two patients indicated they lived with family.

### Task Completion

All 5 activities were successfully completed by all participants, with the exception that 1 patient did not successfully log into the app. It took an average 1.6 minutes for patients to complete each of the 5 activities. The most time-consuming activity was logging into the account, which took a mean of 3 minutes; however, this mean was greatly affected by 2 outlier participants who took 9.4 and 13.8 minutes to complete the task. Of the 10 remaining participants, 8 completed the task in less than 2 minutes, and 2 completed the task in 2-2.5 minutes. The RA observed that some participants took longer to complete the login task because of problems not directly related to the software. For example, 1 patient participant engaged in conversation while attempting to log in, which prolonged the process. Another participant entered an incorrect test password, resulting in failed login attempts. In another instance, a slow-performing phone impacted and slowed the process and appeared to create login failures. The second most time-demanding activity was sending and checking messages (1.9 minutes on average). For clinicians, it took an average 1.4 minutes to complete each of the 5 activities. The most time-demanding activities were changing a patient’s video regimen time and number of uploads and reviewing 1 patient video (both 2.2 minutes on average).

### Workload (NASA-TLX)

Overall, patients and counselors reported low mental, physical, and temporal demands, successful performance, low effort, and low frustration associated with activities. However, counselors reported somewhat higher demands across all categories and activities. Results for the 2 video DOT adherence subgroups and counselors are presented in Tables 2 and 3.

**Table 2:** Activities (NASA Task Load Index) for patients (n=12).

	Task 1: log into account	Task 2: symptom screening completion	Task 3: upload a video mimicking ingestion of methadone	Task 4: send and check a message	Task 5: check progress in the adherence calendar, and state percentage of videos uploaded
Number of people who completed the task successfully^a^, n (%)	11 (92)	12 (100)	12 (100)	12 (100)	12 (100)
Time to complete task (seconds), mean (SD)	181 (251)	50 (38)	80.7 (65)	115 (88)	62.8 (71)
Mental demand (from 0 to 100): “How mentally demanding was the activity?” mean (SD)	6 (11)	5 (11)	10 (15)	6.5 (7)	9.2 (16)
Physical demand (from 0 to 100); “How physically demanding was the activity?” mean (SD)	3.6 (8)	3 (7)	6.5 (6)	2.7 (3)	5.9 (17)
Temporal demand (from 0 to 100): “How hurried or rushed was the pace of the activity?” mean (SD)	7.6 (15)	9.7 (19)	8.2 (15)	5.9 (14)	9.6 (19)
Performance (from 0 to 100): “How successful were you in accomplishing what you were asked to do?” mean (SD)	81.2 (37)	97.5 (7)	97.5 (6)	90.3 (28)	94 (17)
Effort (from 0 to 100): “How hard did you have to work to accomplish your level of performance?” mean (SD	6 (14)	7.5 (20)	13.3 (25)	4.7 (5)	8.8 (23)
Frustration (from 0 to 100): “How insecure, discouraged, irritated, stressed, and annoyed were you?” mean (SD)	4 (8)	4.75 (14)	6.7 (8)	4.5 (7)	1.6 (3)

^a^Task 1 successful completion: logging into the mobile app using the username and password provided. Task 2 successful completion: indicating both cough and fever on the symptom screener. Task 3 successful completion: recording video with all instructions followed and submitting video. Task 4 successful completion: checking the message and replying to the question in the mobile app chat function. Task 5 successful completion: locating the adherence calendar and stating the percentage.

**Table 3:** Activities (NASA Task Load Index) for counselors (n=3).

	Task 1: adding a new patient	Task 2: reviewing 1 patient video	Task 3: changing a patient’s regimen time and number of uploads	Task 4: sending and checking a message	Task 5: checking the patient “adherence calendar” and stating percentage of adherent video
Number of people completed task successfully, n (%)	2 (67)	3 (100)	1 (33)	2 (67)	1 (33)
Time to complete task (seconds), median (min, max)	99 (73, 144)	128 (53, 213)	95 (59, 247)	91 (27, 108)	71 (68, 77)
Mental demand (from 0 to 100): “How mentally demanding was the activity?” median (min, max)	25 (10, 60)	60 (10, 75)	50 (30, 70)	15 (5, 60)	69 (5, 70)
Physical demand (from 0 to 100): “How physically demanding was the activity?” median (min, max)	25 (1, 50)	10 (1, 50)	30 (1, 50)	5 (1, 50)	5 (0, 50)
Temporal demand (from 0 to 100): “How hurried or rushed was the pace of the activity?” median (min, max)	10 (1, 50)	10 (1, 40)	30 (1, 50)	1 (0, 50)	5 (1, 50)
Performance (from 0 to 100): “How successful were you in accomplishing what you were asked to do?” median (min, max)	25 (0, 100)	50 (40, 75)	50 (0, 90)	100 (0, 100)	0 (0, 100)
Effort (from 0 to 100): “How hard did you have to work to accomplish your level of performance?” median (min, max)	25 (10, 70)	25 (25, 60)	60 (30, 75)	15 (5, 75)	50 (20, 50)
Frustration (from 0 to 100): “How insecure, discouraged, irritated, stressed, and annoyed were you?” median (min, max)	26 (15, 60)	60 (25, 75)	60 (50, 75)	10 (5, 60)	60 (10, 90)

### Usability (SUS)

Mean SUS scores reflected high usability for patients (mean 85, SD 9.5). Usability was also high for the patient subgroups with low to moderate video DOT adherence (mean 87.5, SD 7.9) and high adherence (mean 79.3, SD 11.2; Table 4), a nominal difference that was not statistically significant (mean difference 8.5, 95% CI –3.8 to 20.8). In contrast, usability ratings for counselors were considerably lower (mean 43.3, SD 17.7).

**Table 4:** Usability score for patients overall (n=12) and by adherence to the app group and counselors (n=3).

Questions	Patients (n=12), mean (SD)	Patients with low to moderate adherence to video DOT^a^ during clinical pilot (n=8), mean (SD)	Patients with high adherence to video DOT during clinical pilot (n=4), mean (SD)	Counselors (n=3), mean (SD)
“I think I would like to use this mobile app (web portal) frequently along with my methadone treatment” (scored from 1 to 5)	4.3 (1)	4.6 (0.7)	3.7 (1.5)	2.3 (0.5)
“I found the mobile app (web portal) unnecessarily complex” (scored from 1 to 5)	1.6 (0.5)	1.3 (0.5)	2 (0)	2.6 (1.1)
“I thought the mobile app (web portal) was easy to use” (scored from 1 to 5)	4.3 (1.2)	4.2 (1.4)	4.2 (0.5)	3 (1)
“I think that I would need the support of a technical person to be able to use this mobile app (web portal)” (scored from 1 to 5)	1.9 (1)	2 (1.3)	1.7 (0.5)	4.3 (0.6)
“I found the various functions in this mobile app (web portal) were well integrated” (scored from 1 to 5)	4.5 (0.7)	4.7 (0.5)	4 (0.8)	3.3 (1.1)
“I thought there was too much inconsistency in this mobile app (web portal)” (scored from 1 to 5)	1.5 (0.5)	1.4 (0.5)	1.7 (0.5)	2.3 (0.6)
“I would imagine that most people would learn to use this mobile app (web portal) very quickly” (scored from 1 to 5)	4.7 (0.5)	4.7 (0.4)	4.5 (0.5)	2 (1)
“I found the mobile app (web portal) very awkward to use” (scored from 1 to 5)	1.5 (0.5)	1.5 (0.5)	1.5 (0.6)	2.6 (1.1)
“I felt very confident using the mobile app (web portal)” (scored from 1 to 5)	4.6 (0.7)	4.7 (0.5)	4.2 (0.9)	2.6 (1.1)
“I needed to learn a lot of things before I could get going with this mobile app (web portal)” (scored from 1 to 5)	3.9 (0.8)	1.8 (1)	2 (0)	4 (1)
Overall SUS^b^ score on a 0 to 100 normalized scale	85 (9.5)	87.5 (7.9)^c^	79.3 (11.2)^c^	43.3 (17.7)

^a^DOT: direct observed therapy.

^b^SUS: System Usability Scale.

^c^The mean difference between the 2 groups of users was 8.5 (95% CI –3.804 to 20.804).

### User engagement (UES-SF)

User engagement was high for patients (mean 3.8, SD 1.1). User engagement was high for the low to moderate video DOT adherence subgroup (mean 3.9, SD 1.2), but it was lower for the high adherence subgroup (mean 2.8, SD 1.1), a nominal difference that was not statistically significant (mean difference 1.1, 95% CI –0.5 to 2.7). Results for specific domain categories are described in Table 5. For counselors, engagement was lower (mean 2.8, SD 0.6), particularly for the reward and perceived usability domains.

**Table 5:** Four domains of the User Engagement Scale–Short Form for patients overall (n=12), by adherence to the app group, and for counselors (n=3).

Questions and subsequent domain scored from 1 to 5	Patients (n=12), mean (SD)	Patients with low to moderate adherence to video DOT^a^ during clinical pilot (n=8), mean (SD)	Patients with high adherence to video DOT during clinical pilot (n=4), mean (SD)	Difference between 2 patient groups of users, mean (95% CI)	Counselors (n=3), mean (SD)
1. “I lost myself in this experience”	2 (0.7)	2 (0.8)	4 (0.8)	N/A^b^	3.7 (0.58)
2. “The time I spent using mobile app (web portal) just slipped away”	2.8 (1.1)	2.9 (1.2)	3.3 (1)	N/A	4 (0)
3. “I was absorbed in this experience”	3.8 (1.3)	3.6 (1.5)	2 (0.8)	N/A	2.3 (0.58)
Mean of items 1-3, measuring the “focused attention” domain. Items scored as the following: strongly disagree=1, disagree=2, neither disagree nor agree=3, agree=4, and strongly agree=5.	2.9 (0.7)	2.8 (0.9)	3.1 (0.3)	0.3 (–0.752 to 1.352)	3.3 (0)
4. “I felt frustrated while using this mobile app (web portal)”	4.4 (0.67)	4.6 (0.5)	4 (0.8)	N/A	2.3 (0.58)
5. “I found this mobile app (web portal) confusing to use””	4.2 (1.14)	4.3 (1.4)	4.3 (0.5)	N/A	3 (1)
6. “Using this mobile app (web portal) was taxing”	4.2 (0.87)	4.6 (0.5)	3.5 (1)	N/A	2 (0)
Mean of items 4-6, measuring the “perceived usability” domain. Scored as the following: strongly disagree=5, disagree=4, neither disagree nor agree=3, agree=2, and strongly agree=1.	4.3 (0.63)	4.5 (0.5)	3.9 (0.7)	0.6 (–0.174 to 1.374)	2.4 (0.51)
7. “This mobile app was (web portal) attractive”	3.5 (0.67)	3.5 (0.8)	2.5 (0.6)	N/A	3 (1)
8. “This mobile app (web portal) was aesthetically appealing”	3.7 (0.78)	3.4 (0.7)	1.8 (0.5)	N/A	2.6 (1.53)
9. “This mobile app (web portal) appealed to my visual senses”	3.3 (0.89)	3.3 (0.9)	2.5 (1)	N/A	3.3 (1.53)
Mean of items 7-9, measuring the “aesthetic appeal” domain. Scored as the following: strongly disagree=1, disagree=2, neither disagree nor agree=3, agree=4, and strongly agree=5	3.5 (0.7)	3.4 (0.8)	2.3 (0.6)	1.1 (0.083 to 2.117)	3 (1.33)
10. “Using this mobile app (web portal) was worthwhile”	4.6 (0.51)	4.8 (0.5)	1.8 (0.5)	N/A	2.7 (1.15)
11. “My experience was rewarding”	4.7 (0.65)	4.9 (0.4)	1.8 (1)	N/A	2.7 (0.58)
12. “I felt interested in this experience”	4.2 (0.62)	4.5 (0.5)	2.3 (0.5)	N/A	2 (0)
Mean of items 10-12, measuring the “reward” domain. Scored as the following: strongly disagree=1, disagree=2, neither disagree nor agree=3, agree=4, and strongly agree=5	4.5 (0.5)	4.7 (0.4)	1.9 (0.6)	2.8 (2.16 to 3.44)	2.4 (0.51)
Total score for user engagement (an average score higher than 3.5 indicates high engagement)	3.8 (1.12)	3.9 (1.2)	2.8 (1.1)	1.1 (–0.498 to 2.698)	2.8 (0.63)

^a^DOT: direct observed therapy.

^b^N/A: not applicable.

## Discussion

### Overview

Methadone is a life-saving medication for patients with OUD, but requirements for in-person DOT can impose significant barriers to treatment. This study found that an mHealth platform that facilitates video-based DOT and COVID-19 symptom screening through smartphones required low workload and had high usability and engagement for patients with OUD receiving methadone and required low workload and had moderate usability and engagement for methadone treatment counselors. However, counselors scored lower across all instruments. Results indicated that the 5 most critical functions of the app could almost always be completed by patients and counselors and that these tasks were associated with low cognitive workload, high usability, and high user engagement, including for patients with low to moderate adherence and high adherence in the original pilot.

Results suggest that video DOT can be usable for patients with OUD in methadone treatment. The strong performance observed for the study cohort, including in patients with low to moderate adherence in the original pilot, suggests that usability was unlikely to be a significant barrier to adherence with video DOT and that other barriers may have contributed more to variability in video DOT adherence. Contextual factors influencing experiences using the app are currently being explored in a separate qualitative study.

Further investigation is necessary to determine the reasons behind counselors’ lower ratings of the mHealth platform’s usability and engagement compared to patients. It is worth noting that the counselor-facing portal had different features than the patient-facing app. For example, the tasks of counselors were different from patients, as patients submitted videos that counselors then reviewed and approved. We speculate that counselor usability ratings could have been impacted by software and clinical workflow issues. These factors need further investigation, especially given the small sample size in our study. However, possible solutions to address these issues could include providing additional training and support to counselors to enable them to feel ownership, ease, and mastery of their role, such as adding more advanced features or customization options to the platform and conducting more rigorous testing and evaluation of the counselor-facing portal to identify and address any specific usability issues. Future work could also examine how to improve the integration of video DOT with the counselors’ clinic routines and existing workloads, including by identifying ways to minimize the potential impacts of such systems on their clinical routines.

Our study has limitations, such as the small sample size, especially for counselors. With 2 of 5 counselors missing, the findings might be unrepresentative and biased. Although descriptive analyses can be conducted with small sample sizes, the precision of results is limited, and we could not perform subgroup analyses to evaluate usability within important patient subgroups (eg, patients experiencing homelessness or higher-severity OUD). There may have been sampling bias, as we were only able to recruit patients and counselors who were in the original pilot program and were still in the clinic over 1 year later. We also recruited only 2 patients with low adherence in the original clinical pilot, which may introduce a bias toward more favorable results, as one would hypothesize that patients with low adherence might be more likely to experience problems with usability (however, several usability ratings were nominally higher for participants with low to medium adherence compared to patients with high adherence). Our study also has strengths, including its focus on analyzing a novel method for DOT for methadone and usability testing with people who may often be overlooked in technology development efforts.

### Conclusion

Little knowledge exists on the usability of mHealth apps for patients and counselors in methadone treatment. This study narrows the knowledge gap by providing information on workload, usability, and engagement with an mHealth app delivered through smartphones for video observation of methadone home dosing and COVID-19 symptom screening. The study demonstrated that a mHealth app to facilitate video-based DOT of methadone was unlikely to create a heavy workload for patients and counselors. Furthermore, there were no trends to suggest that adherence to the app in the original clinical pilot was related to workload, usability, or engagement, indicating that factors unrelated to usability may have impacted adherence when the app was used during the clinical pilot. Although the app was well received by patients, the study highlights opportunities to improve and further investigate usability for counselors, perhaps by improving training or care integration.  

## Abbreviations

DOT: direct observed therapy
FDA: Food and Drug Administration
mHealth: mobile health
NASA-TLX: NASA Task Load Index
OTP: opioid treatment program
OUD: opioid use disorder
RA: research assistant
SUS: System Usability Scale
UES-SF: User Engagement Scale–Short Form
